# A Support Vector Learning-Based Particle Filter Scheme for Target Localization in Communication-Constrained Underwater Acoustic Sensor Networks

**DOI:** 10.3390/s18010008

**Published:** 2017-12-21

**Authors:** Xinbin Li, Chenglin Zhang, Lei Yan, Song Han, Xinping Guan

**Affiliations:** 1 Institute of Electrical Engineering, Yanshan University, Qinhuangdao 066004, China; clzhang@stumail.ysu.edu.cn (C.Z.); lyan@stumail.ysu.edu.cn (L.Y.); hansongysu@sina.cn (S.H.); xpguan@sjtu.edu.cn (X.G.); 2 School of Electronic Information and Electrical Engineering, Shanghai Jiaotong University, Shanghai 200030, China

**Keywords:** target, localization, particle filter, support vector learning, underwater acoustic sensor networks (UASNs)

## Abstract

Target localization, which aims to estimate the location of an unknown target, is one of the key issues in applications of underwater acoustic sensor networks (UASNs). However, the constrained property of an underwater environment, such as restricted communication capacity of sensor nodes and sensing noises, makes target localization a challenging problem. This paper relies on fractional sensor nodes to formulate a support vector learning-based particle filter algorithm for the localization problem in communication-constrained underwater acoustic sensor networks. A node-selection strategy is exploited to pick fractional sensor nodes with short-distance pattern to participate in the sensing process at each time frame. Subsequently, we propose a least-square support vector regression (LSSVR)-based observation function, through which an iterative regression strategy is used to deal with the distorted data caused by sensing noises, to improve the observation accuracy. At the same time, we integrate the observation to formulate the likelihood function, which effectively update the weights of particles. Thus, the particle effectiveness is enhanced to avoid “particle degeneracy” problem and improve localization accuracy. In order to validate the performance of the proposed localization algorithm, two different noise scenarios are investigated. The simulation results show that the proposed localization algorithm can efficiently improve the localization accuracy. In addition, the node-selection strategy can effectively select the subset of sensor nodes to improve the communication efficiency of the sensor network.

## 1. Introduction

In recent years, underwater acoustic sensor networks (UASNs) have been proposed to explore the ocean and realize aquatic applications, such as safety systems, oil platform monitoring, navigation [1,2]. In [3,4], the UASNs are used for the protection of offshore platforms and energy plants. In UASNs, the target localization, which aims to estimate the location of unknown target, is an important task. In advance, the target localization systems are divided into two categories: passive localization [5,6,7] and active localization [8,9]. For instance, a passive localization was presented in [10] to analyse the received acoustic signals based on Energy Detection and extended Kalman Filter (EKF) algorithm. In [11], an optimal long-term robot motion planning algorithm was proposed to realize the active source localization. In addition, an asynchronous localization algorithm with mobility prediction in [12] is presented to realize collaboratively localize an underwater target. In this paper, we investigate the active localization system in UASNs, where the target transmits localization request message actively and the receivers of the sensor nodes estimate the distances to the target.

Difficulty of the underwater target localization is the constrained underwater environment. Firstly, the limited bandwidth capacity and limited battery power [13] make long-distance communication pattern inefficient in the process of communicating between target and sensor nodes.To optimize the limited communication capacity of sensor nodes, the sensor nodes were divided into several subnetworks in [14] to realize short-distance transmissions instead of the long-distance, improving the sensing accuracy of each sensor node. However, the short-distance pattern is only implemented in the process of data aggregation rather than the process of communicating between the target and sensor nodes.Thus, how to realize the low-cost and short-distance pattern in the process of communicating between the target and sensor nodes is one of the pivotal issues to be addressed in this paper. Base on the classification property of SVM [15], SVM algorithm was applied to discriminate whether the target lies in the region near the location of a sensor node. Motivated by classification this character, we propose a node-selection strategy to structure a low-cost and communication-efficient sensor networks by selecting fractional sensor nodes within the efficient range from the entire sensor networks to communicate with the target.

Subsequently, sensor networks are required to achieve the underwater target localization task. In [16,17], the state-space approach based on particle filter method has been employed to realize target localization in UASNs. The particle filter recursively estimates the probability density of the unknown target location conditioned on all measurement data up to the current frame. Using a sequential Monte Carlo method, the probability density is represented by a set of random particles with associated weights which are updated by the likelihood function of observation. In [18], the time of arrival (ToA) measurement model which relies on precise time synchronization and the speed of sound was adopted to construct the likelihood function. The time synchronization has been improved in [19,20,21]. However, the ToA is vulnerable to the sensing noises from the speed of sound which is affected by many factors such as water temperature, pressure, and salinity [22]. The sensing noises will distort the likelihood function, which makes the particles cannot be weighted accurately and leads to “particle degeneracy” problem. In order to solve this problem, various methods such as human memory model [23], adaptive method [24] and mean-shift method [25] were applied to establish the likelihood function. Specially, learning-based methods have been incorporated into particle filter to deal with sensing noise problem. In [26], the least-square support vector regression (LSSVR) was used to obtain the accurate observation in the noise conditions owe to its black-box model. However, considering that the unique underwater circumstances where the target is occluded by objects results in excessive sensing noise, the method cannot be effective in target localization.

In this paper, we investigate a support vector learning-based particle filter algorithm in communication-efficient UASNs to improve the localization accuracy. A node-selection strategy is first proposed, using support vector machine (SVM) algorithm to train all sensor nodes and judge whether the sensor node locates within the communication-efficient range to the target, to select fractional sensor nodes to participate in the sensing process. Since the node-selection strategy provides the short-distance communication pattern, it has advantages of the communication cost and measurement accuracy compared to the analog long-distance particle filter method [27]. Next, based on the raw data obtained from the selected sensor nodes, a LSSVR-based observation model, where an iterative regression function is proposed to deal with distorted raw data, is established to yield accurate observation against the sensing noises. At the same time, we integrate the observation to formulate the likelihood function of the particle filter. Compared with the ToA-based particle filter [28], where the ToA measurement model was adopted to construct the likelihood function, this approach has a better performance against the sensing noise and effectively update the weights of particles to solve the “particle degeneracy” problem. Based on the above solutions, the communication efficiency and localization accuracy are improved. The main contributions of this paper are threefold.

- A node-selection strategy, where the discrimination criteria is the distance to target so as to realize the short-distance communication, is proposed to select fractional number of sensor nodes from the sensor networks. The pattern where less sensor nodes participate in the sensing process by the way of short-distance communication enhances the communication property and reduces the sensing noises.
- A learning-based observation model coupled with an iterative regression function is proposed to yield an accurate observation against the sensing noise.
- A likelihood function integrating the accurate observation is formulated to effectively update the weights of particles, avoiding the “particle degeneracy”. The solution yields an accurate localization result.

The rest of this paper is organized as follows. In Section 2, the problems for particle filter estimation in UASNs are formulated. In Section 3, the detailed solutions for the problems are presented. Then, Section 4 shows the simulation results and analysis of them. Finally, conclusions are given in Section 5.

## 2. Problem Formulation

In this section, we formulate the problems for particle filter estimation in UASNs. First, the network architecture for UASNs is briefly introduced. Next, considering the limited communication resources and sensing noises, the particle filter localization problems are described.

### 2.1. System Model

In [3], the protection system requires a unified model based on aerial, ground and underwater sensor to guarantee foolproof protection. The project targets obtain sensor data using the acoustic communication network. This data is also used to tune the communication system for optimal performance. The tuning process can be carried out by an Autonomous Underwater Vehicle (AUV) to physically separate the acoustic modules and guarantee the time synchronization of the sensor network.

**Figure 1:**
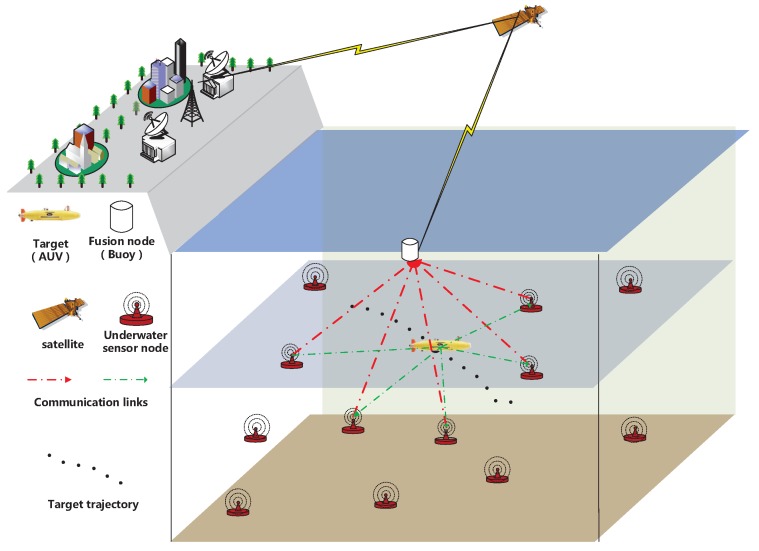
The communication-efficient architecture for underwater acoustic sensor networks.

In this paper, the underwater sensor network is used to realize active localization. In the process of the active localization, the target transmits localization request message and the receivers of the sensor nodes which are attached to the entire monitoring region including its bottom estimate the distances to the target. After this process of sensing, each sensor node obtains the distance data to target. Next, the all distance data is transmitted to fusion node which is capable of data aggregation and computation. As shown in the Figure 1, the AUV is regarded as the target; The buoy floats at the surface of water to aggregate measurement data and realize the communication between underwater and the terrestrial workstation.

In the sensing process, the time-synchronous measurement model of time of arrival (ToA) is adapted to obtain the distance data. Assuming a target and *N* sensor nodes in UASNs, the discrete-time signal received at the *l*th sensor node (where l=1,…,N) is

(1) $$r_{l}=\left(t_{l2}-t_{l1}\right)\cdot v_{s}$$

where $r_{l}$ is the relative distance from target to lth sensor node and $t_{l1}$ is the time that the target sends signal, while the time of receiving signal from the target is $t_{l2}$; $v_{s}\approx 1500$ m/s represents the propagation speed of the acoustic wave.

The acquired distance data from *N* sensor nodes at time frame *k* are stacked to form the raw data $R_{k}$, that is N×1 matrix

(2) $$\begin{array}{c} R_{k}=\left[\begin{array}{c} r_{1}\left(k\right)\\ \vdots \\ r_{N}\left(k\right) \end{array}\right] \end{array}$$

Physically, the observation of target location $Z_{k}$ is obtained through the transformation of the raw data

(3) $$Z_{k}=f\left(\theta ,R_{k}\right)$$

where f(·) is relevant observation function and θ is the observation parameter corresponding to the observation function.

### 2.2. The Problems of the Particle Filter Localization Scheme in UASNs

The state-space model contains the state transition equation and observation equation [29]. At time frame *k*, the mobile target state is described with the state vector $X_{k}={\left[x_{k},y_{k},z_{k}\right]}^{T}$. The state vector can be acquired based on the state transition relation

(4) $$X_{k}=H\left(X_{k-1},v_{k-1}\right)$$

where H(·) is a known, not necessarily linear, function of the previous state $X_{k-1}$ and a noise term $v_{k-1}$. The observation state-space equation is described as

(5) $$Z_{k}=F\left(X_{k},w_{k}\right)$$

where F(·) is an unknown, not necessarily linear, function of the state $X_{k}$ and a noise term $w_{k}$.

**Problem** **1.** *As described in Section 2.1, the raw data is obtained from N sensor nodes at time frame k. However, in underwater region, the sensor nodes staying away from the target are unable to communicate with the target effectively because of the limited communication capacity. Moreover, it is known that long-distance communication means more noises and the obtained distance data from the sensor node staying away from target is not available to the fusion node. Thus, we prefer short-distance transmissions that are influenced by less noises compared with long-distance transmissions in an underwater environment. Based on this, we propose a communication-efficient network architecture shown in Figure 1. In the sensor network, the fractional sensor nodes coming close to the target are selected to transmit their distance data to fusion node.*

Let $Z_{1:k}=\left\{Z_{1},Z_{2},\ldots ,Z_{k}\right\}$ denotes all observations up to time *k*. The aim is to recursively estimate the posterior probability density function, that is, $p(X_{k}|Z_{1:k})$. Based on Bayesian recursive estimation, the posterior distribution [30] can be described as

(6) $$p\left(X_{k}|Z_{1:k-1}\right)=\int p\left(X_{k}|X_{k-1}\right)p\left(X_{k-1}|Z_{1:k-1}\right)$$

(7) $$p\left(X_{k}|Z_{1:k}\right)\propto p\left(Z_{k}|X_{k}\right)p\left(X_{k}|Z_{1:k-1}\right)$$

where $p(X_{k-1}|Z_{1:k-1})$ is the posterior distribution estimated at the last time step, and $p(X_{k}|Z_{1:k-1})$ is the prior distribution for the current time step. Equation (4) is modeled as a state transition probability $p(X_{k}|X_{k-1})$ and the Equation (5) is modeled as the likelihood function $p(Z_{k}|X_{k})$.

In general, the Bayesian recursion described in (6) and (7) cannot be calculated analytically except for special linear Gaussian state-space model such as Kalman filter. For the nonlinear localization model, the particle filter approximates the posterior probability function using Monte Carlo method to sample a set of particles associated with weights [31]. Let ${\left\{x_{k}^{i},w_{k}^{i}\right\}}_{i=1}^{N_{p}}$ denotes $N_{p}$ weighted particles. Thus, (7) is represented as follows

(8) $$p\left(X_{k}|Z_{1:k}\right)≃\sum_{i=1}^{N_{p}}w_{k}^{i}\delta \left(X_{k}-x_{k}^{i}\right)$$

where δ(·) is the Dirac delta function. Considering the difficulty of drawing the particles from (8), the important sampling is used to draw the particles using known important distribution function $q(X_{k}|X_{1:k-1},Z_{1:k})$. By employing the posterior distribution function as the important distribution function, that is $q\left(X_{k}|X_{1:k-1},Z_{1:k}\right)=p\left(X_{k}|X_{k-1}\right)$, the particles and weights are given as

(9) $$x_{k}^{i}∼p\left(X_{k}|X_{k-1}\right)$$

(10) $$w_{k}^{i}=w_{k-1}^{i}\frac{p\left(Z_{k}|x_{k}^{i}\right)p\left(x_{k}^{i}|x_{k-1}^{i}\right)}{q(X_{k}|X_{1:k-1},Z_{1:k})}=w_{k-1}^{i}p\left(Z_{k}|x_{k}^{i}\right)$$

The likelihood function is designed as Gaussian likelihood function given by

(11) $$p\left(Z_{k}|x_{k}^{i}\right)=\prod_{j\in M}N\left(Z_{k}^{j};d_{k}^{j},\sigma \right)$$

where $N(Z_{k}^{j},d_{k}^{j},\sigma )$ represents a Gaussian distribution with mean $d_{k}^{j}$ and variance σ; $d_{k}^{j}=∥x_{k}^{i}-s^{j}∥$ is the distance between the *i*th particle and *j*th selected sensor node (j∈M). $s^{j}$ is the known location of *j*th sensor node.

Thus, the target location can be estimated as

(12) $${\hat{X}}_{k}=\sum_{i=1}^{N_{p}}w_{k}^{i}x_{k}^{i}$$

**Figure 2:**
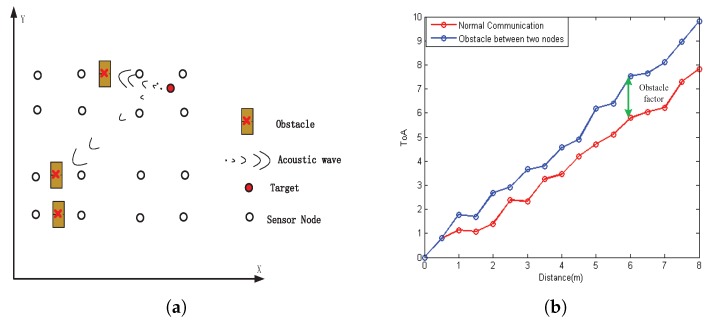
(**a**) The target is occluded by obstacle; (**b**) ToA measurement in the situation of obstacle between two nodes.

**Problem** **2.** *The Equation (10) reveals that the weights are updated by likelihood function $p(Z_{k}|x_{k}^{i})$. The measurement model (1) is easily influenced by the complex underwater environment. Many kinds of sensing noises are introduced into raw data $R_{k}$ at time frame k. The inaccurate acoustic propagation is a vital factor. In general, the sound propagation $v_{s}$ is closely connected with the density of the water, the temperature of the water, the multipath and so on. Consequently, the unauthentic propagation speed results in sensing noise. Moreover, as shown in Figure 2a, the line of sight between target and sensor node is blocked by the appearance of the obstacles. The relationship between ToA measurement and distance is shown in Figure 2b, which reveals the excessive sensing noise is contained in the raw data $R_{k}$ when the acoustic link is blocked by the obstacles. When the raw data is distorted, we mark it as $Z_{k}^{j*}$. When $Z_{k}^{j*}>Z_{k}^{j}$ is established, it is obvious that the inequality $\omega_{k}^{j*}<\omega_{k}^{j}$ is approved. Thus, the distorted raw data will lead to the problem of “particle degeneracy”, where the particles can obtain negligible weights after some updates. Accordingly, the estimated target location will lose accuracy.*

## 3. Algorithm Description

In this section, we first design a node-selection strategy, selecting appropriate sensor nodes from the underwater sensor network, to improve the communication efficiency. Based on the optimized sensor network, the support vector learning-based particle filter method is then developed to improve the localization accuracy.

### 3.1. Node-Selection Strategy for UASNs

As described in **Problem 1**, it is unreasonable that the fusion node gathers the data from each sensor node. Thus, we select fractional sensor nodes locating within efficient range ς to the target to transmit their distance data to fusion node. A node-selection strategy is designed to ascertain the satisfactory sensor nodes, using SVM algorithm to train all sensor nodes.

We consider a sensor network with *N* sensor nodes, each of which obtains distance data $r_{l}\left(k\right)$ at time frame *k*, l=1,…,N. Given a training dataset ${\left\{d_{i},y_{i}\right\}}_{i=1}^{N_{s}}$. $N_{s}$ denotes the total number of training data and $y_{i}\in \left\{-1,1\right\}$ is the label corresponding to $d_{i}$ ($0\leq d_{i}\leq \vartheta$). Each sensor node can be trained by solving the following dual form of the SVM problem [8]

(13) $$\begin{array}{c} \min_{\lambda_{i}}\frac{1}{2}\sum_{i,j}y_{i}y_{j}\lambda_{i}\lambda_{j}\varphi \left(d_{i},d_{j}\right)-\sum_{i}\lambda_{i}\\ s.t.\sum_{i}\lambda_{i}y_{i}=0,\;0\leq \lambda_{i}\leq P,\;i,j=1,\ldots N_{s} \end{array}$$

where $\lambda_{i}$ is the corresponding Lagrangian multipliers, *P* is a regularization constant and and φ(·,·) is a kernel function to express the nonlinearity of the data distribution. Solving the optimization problem (13) with quadratic programming, a discriminant function is obtained as

(14) $$f^{\left(l\right)}\left(r_{l}\left(k\right)\right)=\sum_{i=1}^{N_{s}}\lambda_{i}y_{i}\varphi \left(r_{l}\left(k\right),d_{i}\right)$$

The discriminant (14) decides whether *l*th sensor node within the efficient range ς $\left(f^{\left(l\right)}\left(r_{l}\left(k\right)\right)>0\right)$ or not $\left(f^{\left(l\right)}\left(r_{l}\left(k\right)\right)<0\right)$. Based on this node-selection strategy, we pick out *M* sensor node from the entire sensor network.

**Remark** **1.** *Based on the node-selection strategy, the less sensor nodes are used to participate in the transmission process by means of short-distance communication. Thus, the communication efficiency will be improve at every transmission frame and the short-distance transmissions mean less influence of sensing noise, such that fusion node can obtain more accurate raw data. Meanwhile, the energy consumption of the sensor network reduce owe to less transmissions process.*

### 3.2. Support Vector Learning-Based Particle Filter Method

In this section, we focus on designing a support vector learning-based particle filter method base on the aforementioned sensor network to solve the **Problem 2**.

#### 3.2.1. Least-Square Support Vector Regression (LSSVR)-based Observation Function

The traditional particle filter regards the raw data as observation, that is $Z_{k}=R_{k}$. The raw data is easily distorted by the underwater noises, which leads to the likelihood function cannot be formulated efficiently. In order to obtain accurate observation in the condition of sensing noises, we first establish LSSVR-based observation model. Consider a regression problem using a training dataset $D={\left\{x_{m},y_{m}\right\}}_{m=1}^{J}$, where *J* denotes the total number of training data, $x_{m}\in R^{M}$ is the input pattern with the selected *M* sensor nodes, and $y_{m}$ is the corresponding output. A nonlinear observation function can be obtained by solving the following optimization problem [32]:

(15) $$\begin{array}{c} \min_{\omega ,e}\left\{\frac{1}{2}\omega^{T}\omega +\frac{C}{2}\sum_{m=1}^{J}e_{m}^{2}\right\}\\ s.t.\;y_{m}=\omega^{T}\phi \left(x_{m}\right)+b+e_{m},\;m=1,\ldots J \end{array}$$

where the vector ω represents the model complexity, b is the bias. $e={\left[e_{1}\ldots e_{J}\right]}^{T}$ and C≥0 are the margin and the gain of the regulator, respectively. ϕ(·) is a nonlinear mapping that maps the input data into a high-dimensional feature space whose dimensions can be infinite. As the Lagrange is applied to (15), we get the following form:

(16) $$L\left(\omega ,b:e,\alpha \right)=\sum_{m=1}^{J}\alpha_{m}\left(y_{m}-\omega^{T}\phi \left(x_{m}\right)-b-e_{m}\right)+\frac{1}{2}\omega^{T}\omega +\frac{C}{2}\sum_{m=1}^{J}e_{m}^{2}$$

where $\alpha ={\left[\alpha_{1},\ldots \alpha_{J}\right]}^{T}$ is the Lagrange multiplier that can be either positive or negative. Based on the Karush-Kuhn-Tucker (KKT) condition, the (16) is replaced by the following matrix equation

(17) $$\begin{array}{c} \left[\begin{array}{cc} 0 & \bar{1}\\ \bar{1} & \bar{K} \end{array}\right]\left[\begin{array}{c} b\\ \alpha \end{array}\right]=\left[\begin{array}{c} 0\\ \bar{y} \end{array}\right] \end{array}$$

where $\bar{1}={\left[1_{1},\ldots 1_{J}\right]}^{T}$, $\bar{y}={\left[y_{1},\ldots y_{J}\right]}^{T}$, and $\bar{K}$ is an J×J matrix whose elements are given as

(18) $${\bar{K}}_{mn}=\kappa \left(x_{m},x_{n}\right)=\phi {\left(x_{m}\right)}^{T}\phi \left(x_{n}\right)+\eta_{mn}/C$$

In (18), $\eta_{mn}$ is given as

(19) $$\begin{array}{c} \eta_{mn}=\left\{\begin{array}{c} 1,m=n\\ 0,m\neq n \end{array}\right. \end{array}$$

After the α and *b* are obtained by solving matrix Equation (17), the observation function is formed as

(20) $$Z_{k}=\sum_{m=1}^{J}\alpha_{m}k\left(x_{m},R_{k}\right)+b$$

Furthermore, in obstacles condition, the obstacles will introduce excessive sensing noise to $R_{k}$. In order to address excessive sensing noise problem, we propose an iterative regression function based on LSSVR to establish the observation function, given by

(21) $$Z_{k}^{\left(\nu +1\right)}=Z_{k}^{\left(\nu \right)}\left(\delta R_{k}\right)=Q\left(Z_{k}^{\left(\nu \right)}\right)$$

where superscript ν denotes the iteration step. As before, $Z_{k}$ values are the predictor; thus, they can be treated as constants throughout the iterations. In Section 3.1, *M* sensor nodes have been selected to transmit their distance data to fusion node, which composes of the M×1 vector data $R_{k}$.

(22) $$\begin{array}{c} R_{k}=\left[\begin{array}{c} r_{1}\left(k\right)\\ \vdots \\ r_{M}\left(k\right) \end{array}\right] \end{array}$$

At the frame *k*, it is assumed that target has its own transmitted intensity level $T_{IL}$ , and $R_{IL}$ denotes the received intensity level of sensor node. In the process of transmitting and receiving, the propagation loss of the acoustic wave PL is denoted. Thus, the received intensity level $R_{IL}$ for an arbitrary sensing sensor is defined as

(23) $$R_{IL}=T_{IL}-PL$$

Under the surface of the water, the propagation loss PL is denoted as follows [33]

(24) $$PL=20\log\hat{r}+\beta \hat{r}\cdot 10^{-3}$$

where β>0 is the attenuation coefficient, and $\hat{r}$ is the relative distance level from target to sensor.

We establish the following diagnosis mechanism of excessive sensing noise to determine the δ, the *l*th element of $R_{k}$ is diagnosed

(25) $$\left\{\begin{array}{c} T_{IL}-R_{IL}\geq \hat{p}\tau \left(R_{k}\left[l\right]\right),0<\hat{p}\leq 1\\ \tau \left(R_{k}\left[l\right]\right)=20\log R_{k}\left[l\right]+\beta R_{k}\left[l\right]\cdot 10^{-3} \end{array}\right.$$

where $R_{k}\left[l\right]$ denotes the *l*th element of the raw data $R_{k}$ and $0<\hat{p}\leq 1$ is the gain of propagation loss. If $T_{IL}-R_{IL}\geq \hat{p}\tau \left(R_{k}\left[l\right]\right)$, it means that the excessive sensing noise is contained in raw data $R_{k}\left[l\right]$.

**Remark** **2.** The gain of propagation loss $\hat{p}$ guarantees the flexibility and practicality because the sensing noise is closely related with the environment and the communication capacity of the sensor nodes.

Based on the diagnosis mechanism of excessive sensing noise, the (l,l)th element of the M×M diagonal matrix δ is given by

(26) $$\delta_{\left(l,l\right)}=\left\{\begin{array}{c} \frac{|R_{k}\left[l\right]-{\bar{S}}_{l}|}{R_{k}\left[l\right]},\;T_{IL}-R_{IL}\geq \hat{p}\tau \left(R_{k}\left[l\right]\right)\\ 1,\;otherwise \end{array}\right.$$

where $\bar{S_{l}}$ is the average of the *l*th element of all data in training dataset *D*.

**Remark** **3.** *Based on the diagnosis mechanism in (25), we can determine whether $R_{k}\left[l\right]$ is distorted or not. Meanwhile, the ${\bar{S}}_{l}$ is determined by the data from dataset D. When the distorted $R_{k}\left[l\right]$ contains excessive sensing noise, the corresponding $\delta_{\left(l,l\right)}$ will be smaller. This characteristic makes the distorted data be addressed more efficient. After δ is obtained, the iterative regression function (21) is used to estimate the final observation $Z_{k}$.*

In general, the termination condition is indicated as $\left|Z_{k}^{\left(\nu +1\right)}-Z_{k}^{\left(\nu \right)}\right|\leq \varepsilon$. However, the (21) will be approximately equal to *b* which is obtained by solving the matrix Equation (17) in the initial iteration steps, when the sensing noises contained in raw data $R_{x}$ are large enough. Namely, it reaches the termination condition prematurely. Thus, we propose the following termination form:

(27) $$\left\{\begin{array}{c} |Z_{k}^{\left(\nu +1\right)}-b|\geq \epsilon \\ |Z_{k}^{\left(\nu +1\right)}-Z_{k}^{\left(\nu \right)}|\leq \varepsilon \end{array}\right.$$

where ϵ and ε are positive constants. $\left|Z_{k}^{\left(\nu +1\right)}-b\right|\geq \epsilon$ can avoid reaching the termination condition prematurely and $\left|Z_{k}^{\left(\nu +1\right)}-Z_{k}^{\left(\nu \right)}\right|\leq \varepsilon$ guarantees that the (21) converges to proper value. Thus, the LSSVR-based observation function is shown in Algorithm 1.

**Algorithm 1** LSSVR-based observation function
1:Initialization:Train LSSVR model with training dataset *D* and acquire α and *b* by solving matrix Equation (17)2:Input: Raw data $R_{k}$3:**Diagnosis of excessive sensing noise**4:**for all** l=1,…,M **do**5: **if** There exists $T_{IL}-R_{IL}\geq \hat{p}\tau \left(R_{k}\left[l\right]\right)$ and $\tau \left(R_{k}\left[l\right]\right)=20\log R_{k}\left[l\right]+\beta R_{k}\left[l\right]\cdot 10^{-3}$ in (25) **then**6:  Corresponding parameter which the excessive sensing occurs is set based on (26): $\delta_{\left(l,l\right)}=\frac{|R_{k}\left[l\right]-{\bar{S}}_{l}|}{R_{k}\left[l\right]}$7: **else**8:  Corresponding parameter which the excessive sensing does not occur is set based on (26): $\delta_{\left(l,l\right)}=1$9: **end if**10:**end for**11:All corresponding parameters are determined and the diagonal matrix δ is obtained12:**Iteration: calculate the observations**13:**while** Termination condition: $\left|Z_{k}^{\left(\nu +1\right)}-b\right|\geq \epsilon$ and $\left|Z_{k}^{\left(\nu +1\right)}-Z_{k}^{\left(\nu \right)}\right|\leq \varepsilon$ **do**14: The iterative equation: $Z_{k}^{\left(\nu +1\right)}=Z_{k}^{\left(\nu \right)}\left(\delta R_{k}\right)=Q\left(Z_{k}^{\left(\nu \right)}\right)$, ν=0,1,2…15:**end while**16:Output: Observation of $Z_{k}=Z_{k}^{\left(\nu +1\right)}$

#### 3.2.2. Formulation Likelihood Function

The traditional particle filter regards the raw data as observations. Accordingly, the likelihood function $p(Z_{k}|x_{k}^{i})$ in (10) is used to update the weight $w_{k}^{i}$. The likelihood function is designed as Gaussian likelihood function given by

(28) $$p\left(Z_{k}|x_{k}^{i}\right)=\prod_{j\in M}N\left(Z_{k}^{j};d_{k}^{j},\sigma \right)$$

where $N(Z_{k}^{j},d_{k}^{j},\sigma )$ represents a Gaussian distribution with mean $d_{k}^{j}$ and variance σ; $d_{k}^{j}=∥x_{k}^{i}-s^{j}∥$ is the distance between the *i*th particle and *j*th selected sensor node (j∈M). $s^{j}$ is the known location of *j*th sensor node. Thus, the weight of the *i*th particle is updated

(29) $$w_{k}^{i}=w_{k-1}^{i}\prod_{j\in M}\frac{1}{\sqrt{2\pi }\sigma }exp\left(-\frac{{\left(R_{k}\left[j\right]-d_{k}^{j}\right)}^{2}}{2\sigma^{2}}\right)$$

As described in Section 3.2.1, the LSSVR-based observation function is established to construct the mapping $f\left(\cdot \right):R^{M}⟶R$ from raw data to three-dimensional target location. Once this mapping is determined, the three-dimensional target location is regarded as the observation $Z_{k}$. The likelihood function and weight update are as follows

(30) $$p\left(Z_{k}|x_{k}^{i}\right)=N\left(Z_{k};x_{k}^{i},\sigma \right)$$

and

(31) $$w_{k}^{i}=w_{k-1}^{i}\frac{1}{\sqrt{2\pi }\sigma }exp\left(-\frac{∥Z_{k}-x_{k}^{i}{∥}^{2}}{2\sigma^{2}}\right)$$

The support vector learning-based particle filter algorithm, where the LSSVR-based observation function is used to computer the observation and the proposed likelihood function is used to update the particle weights, is shown in Algorithm 2.

**Algorithm 2** Support vector learning-based particle filter algorithm1:Initialization: generate $N_{p}$ initial particles $\left\{x_{0}^{i},i=1,\ldots N_{p}\right\}$ and give them uniform weights $\left\{w_{0}^{i}=1/N_{p},i=1,\ldots ,N_{p}\right\}$2:**for all** k=1,2… **do**3: **for all** $i=1,2\ldots N_{p}$ **do**4:  - Draw particles according to state transition model (4)5:  $x_{k}^{i}∼p\left(x_{k}|x_{k-1}^{i}\right)$6:  - Compute the observation according to the **Algorithm 1**7:  $Z_{k}=Z_{k}^{\left(\nu +1\right)}$8:  - Compute the likelihood function based on (30): $p\left(Z_{k}|x_{k}^{i}\right)=N\left(Z_{k};x_{k}^{i},\sigma \right)$9:  - Update the weights based on (31): $w_{k}^{i}=w_{k-1}^{i}\frac{1}{\sqrt{2\pi }\sigma }exp\left(-\frac{∥Z_{k}-x_{k}^{i}{∥}^{2}}{2\sigma^{2}}\right)$10: **end for**11: - Obtain total particle weights $w_{k}^{i}\left(i=1,2\ldots N_{p}\right)$12: - Normalize the weight: $w_{k}^{i}=w_{k}^{i}/\sum_{i=1}^{N_{p}}w_{k}^{i}$13: - Resample the particles according to the weights $w_{k}^{i}$:weed out low-weight particles14: - Estimate the target state ${\hat{X}}_{k}=\sum_{i=1}^{N_{p}}w_{k}^{i}x_{k}^{i}$15:**end for**


## 4. Results and Discussion

In this section, we deploy a simulation environment with 20 sensor nodes to a region of 100 m × 100 m × 100 m. First, we investigate the communication-efficient network architecture. According to the Section 3.1, the optimized network architecture in this paper can pick fractional sensor nodes by the node-selection strategy to improve the communication efficiency. The distance parameter is set ϑ=180 to train the monitored region. We set the communication-efficient range as ς = 10 m, 40 m, 50 m, 60 m, 70 m, 80 m and 100 m respectively. When the target locates in (40,50,60), we give the Table 1 to show the discriminant performance of node-selection strategy in the case of different communication-efficient range. According to the Table 1, the node-selection strategy has a great performance in discriminating and selecting sensor nodes in the different communication-efficient range.

**Figure 3:**
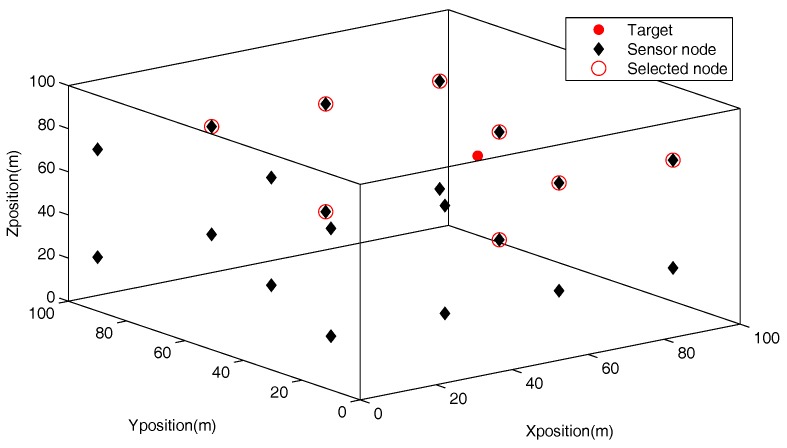
The selected sensor nodes in communication-constrained underwater acoustic sensor networks.

Meanwhile, considering that if the number of selected sensor nodes is too small, the obtained observation in Section 3.2 will degrade because of the lack of data information. Thus, in the following simulated experiments, we set the communication-efficient range as ς=50 m, avoiding the shortage of information. An example with target locating in (51.08,40.95,60.75) is shown in Figure 3. It can be shown that the node-selection strategy select appropriate sensor nodes, that means M=8.

Next, this section is devoted to the experimental study for the verification of the localization performance of the proposed support vector learning-based particle filter (SVL-PF) in sensing noise condition. According to [26,28], the least-square support vector regression and ToA are used to establish the observation function of particle filter respectively, improving the accuracy of the measurement data. In Consensus Estimation [14], a regional optimal solution is proposed to avoid the occurrence of no-solution situation and improve the localization accuracy. These algorithms have been demonstrated to be effective when handling the universal sensing noise. In this paper, we propose a support vector learning-based particle filter (SVL-PF) scheme to solve the excessive sensing noise on the basis of the universal sensing noise. Thus, we compare the proposed SVL-PF algorithm with Consensus Estimation [14], LSSVR-PF [26] and ToA-PF [28] in two different simulated scenarios from both universal sensing noise and excessive sensing noise.

For all the simulated experiments, the gain of propagation loss $\hat{p}$ in (25) is selected to be 1/2. The parameters ϵ and ε in (27) are set to 1 and 0.2, guaranteeing the convergence performance. Since the underwater target is usually assumed to be moving slowly, the CV model is employed for the state model

$$\begin{array}{c} \left[\begin{array}{c} X_{k}\\ {\dot{X}}_{k} \end{array}\right]=\left[\begin{array}{cc} 1 & T\\ 0 & 1 \end{array}\right]\left[\begin{array}{c} X_{k-1}\\ {\dot{X}}_{k-1} \end{array}\right]+\left[\begin{array}{c} 1\\ 0 \end{array}\right]V_{k} \end{array}$$

where *T* representing the time period in seconds between the previous and current time step; ${\dot{X}}_{k}$ is the motion velocity and $X_{k}$ is the target state. $V_{k}$ is a Gaussian random variable with zero mean and unit variance. We set the initial state $X_{0}={\left(1,1,1\right)}^{T}$ and initial particles are drawn uniformly around the workspace.

**A. Scenario 1: A localization example in the universal sensing noise condition**

**Figure 4:**
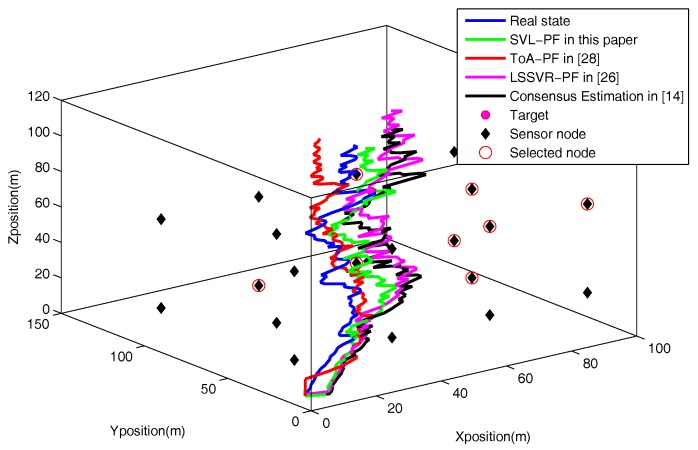
The localization trajectory of the proposed algorithm and the compared algorithms in the universal noise condition.

In this scenario, we check the localization performance in the case where the universal sensing noise is mingled in raw data. A Gaussian noise with a standard deviation of 10% of the raw data is added to the raw data. The sensing noise is mainly caused on account of the insufficient communication ability of sensor node. Figure 4 shows the localization trajectories of four algorithms. In the figure, the green curve (SVL-PF estimation ) preferably follows the blue curve (real state) than the red curve (ToA-PF), black curve (Consensus Estimation) and purplish red curve (LSSVR-PF).

**Figure 5:**
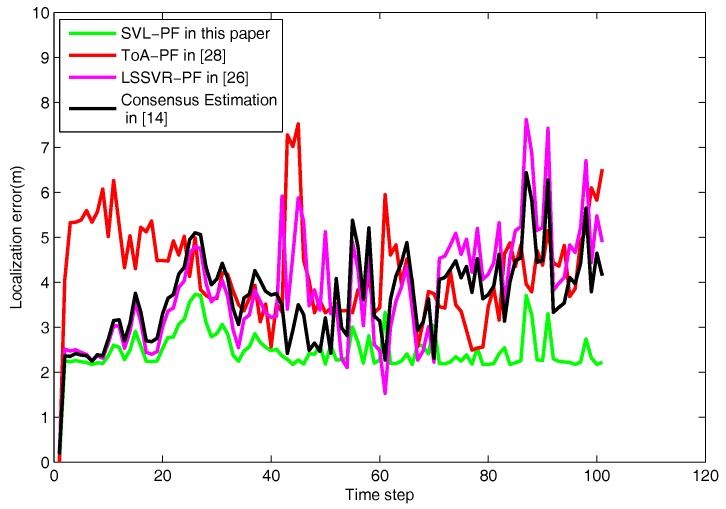
The localization errors of the proposed algorithm and the compared algorithms in the universal noise condition.

To show more clearly, a localization error function is defined as $E_{k}={\left[{\left({\hat{x}}_{k}-x_{k}\right)}^{2}+{\left({\hat{y}}_{k}-y_{k}\right)}^{2}+{\left({\hat{z}}_{k}-z_{k}\right)}^{2}\right]}^{\frac{1}{2}}$, where ${\hat{X}}_{k}={\left({\hat{x}}_{k},{\hat{y}}_{k},{\hat{z}}_{k}\right)}^{T}$ is the estimated location of the target and $X_{k}={\left(x_{k},y_{k},z_{k}\right)}^{T}$ is the real location of the target at time frame *k*. The localization errors using the proposed SVL-PF algorithm in this paper and the compared algorithms are shown in Figure 5.

As shown, even though the localization errors of SVL-PF are larger than the compared algorithms at certain time frames such as k=54, 61 and 66, it is obvious that the SVL-PF presents better localization accuracy as a whole.

Moreover, considering that the particle filter recursively estimates target location conditioned on all measurement data up to the current time frame, the prior results have an effect on the current time frame. Thus, the average of accumulated error function is defined to show the average localization error from the initial time frame to present time frame *k*.

(32) $$E_{ave}=\sum_{k=1}^{t_{k}}E_{k}/k$$

where $E_{k}$ is the localization error at time frame *k* and $t_{k}$ represents the current time frame. When the current time frames are $t_{k}=10,20\ldots ,100$, the average localization errors of all test algorithms are shown in Table 2.

Based on the Table 2, the SVL-PF in this paper has smaller average localization error at different time frames. The significance of the different results among the algorithms (SVL-PF, ToA-PF, LSSVR-PF and Consensus Estimation) illustrates that the SVL-PF using the support vector learning-based measurement model is efficient against the sensing noise.

According to the results, we can easily verify that the proposed SVL-PF algorithm in this paper presents better localization performance, although the compared algorithms have a good localization performance in the universal sensing noise. It means that the proposed algorithm in this paper has a better localization accuracy in the situation of universal sensing noise.

**B. Scenario 2: A localization example in the excessive sensing noise condition**

**Figure 6:**
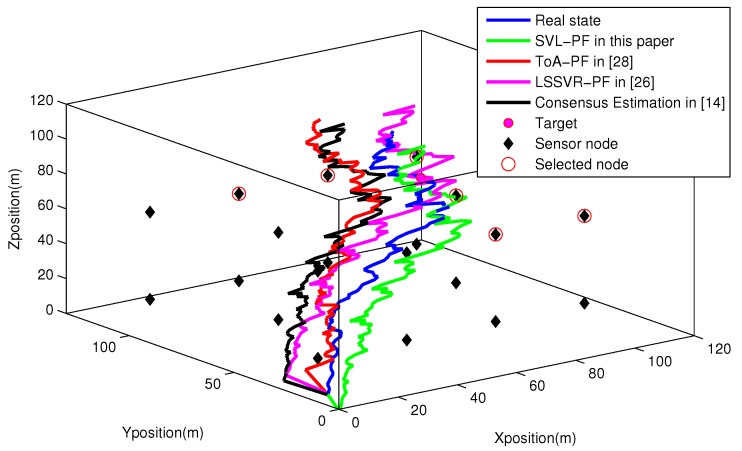
The localization trajectory of the proposed algorithm and the compared algorithms in the excessive noise condition.

**Figure 7:**
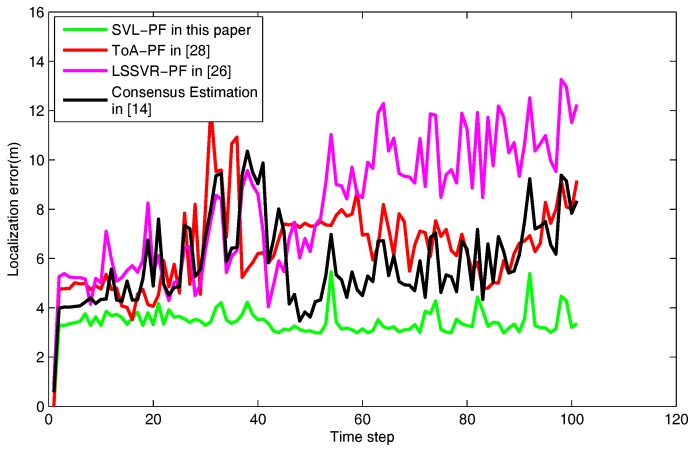
The localization errors of the proposed algorithm and the compared algorithms in the excessive noise condition.

As described in **Problem 2**, the excessive sensing noise will lead to particle degeneracy and accuracy loss. In this scenario, we check the localization performance in the case where the excessive sensing noise arises. On the basis of the universal sensing noise, a Gaussian noise with a standard deviation of 25% of the raw data is additional added to the raw data $R_{k}$. Similarly, the localization trajectory and the corresponding localization errors are described in Figure 6 and Figure 7. Taking Figure 7 as an example, the localization errors of the ToA-PF, LSSVR-PF and Consensus Estimation are larger than the proposed SVL-PF in this paper. Moreover, Table 3 shows the average localization errors of all test algorithms in the excessive sensing noise condition. The compared algorithms (ToA-PF, Consensus Estimation and LSSVR-PF) contain the larger average localization errors which reach to 6.8802 m, 5.9295 m and 8.2197 m. It is obvious that the SVL-PF has a smaller average localization errors from the initial time frame to the final time frame. The results declare that the LSSVR-based and ToA-based cannot effectively handle the excessive sensing noise problem, and the the excessive sensing noise results in the no-solution situation, causing the localization accuracy loss of the Consensus Estimation algorithm. Besides, compared with the Table 2 and Table 3, the average localization error of LSSVR-PF algorithm has a great increase from universal condition to excessive condition. The reason is that the LSSVR model closely related to the raw data cannot obtain satisfactory measurement data in excessive noise condition and the correlation character of particle filter among all measurement data up to the current time frame aggravates the localization accuracy.

As shown, SVL-PF is the most effective algorithm in terms of excessive sensing noise. It demonstrates that SVL-PF can solve the excessive sensing noise problem by means of the iterative regression function and improve the localization accuracy.

It is found from the aforementioned results that the proposed SVL-PF algorithm in this paper has a better performance against the sensing noise from both universal sensing noise and excessive sensing noise. Specially, in the face of the sensing noise, SVL-PF algorithm can solve this problem and improve the localization accuracy.

## 5. Conclusions

In this paper, we present the support vector learning-based particle filter for target localization in communication-constrained underwater acoustic sensor networks. By taking advantage of the node-selection strategy, the pattern where less sensor nodes participate in sensing process by the way of short-distance communication improves the efficiency of communication and reduces the sensing noise. Furthermore, a support vector learning-based particle filter approach formulates the likelihood function to update the weights of particles, and yields accurate estimation in spite of excessive sensing noise condition. We compare our algorithm with the related particle filter-based algorithms and consensus estimation algorithm in estimating the target location. The simulation results show that the node-selection strategy can effectively select the subset of sensor nodes and the proposed localization algorithm can efficiently improve the localization accuracy than particle filter in the condition of sensing noises.

## Figures and Tables

**Table 1:** Discriminant of sensor nodes in different communication-efficient range.

Communication-Efficient Range	10	40	50	60	70	80	90	100
**Number of sensor node within the distance**	0	2	8	14	18	20	20	20
**Discriminant of sensor nodes**	0	2	8	14	18	19	19	19

**Table 2:** The average localization errors in the universal noise condition.

	Time Step (s)	k=10	k=20	k=30	k=40	k=50	k=60	k=70	k=80	k=90	k=100
Method											
SVL-PF in this paper		2.2723	2.3781	2.6469	2.6243	2.5688	2.5631	2.5405	2.5059	2.5209	2.4971
Consensus Estimation in [14]		2.4758	2.7938	3.3335	3.4380	3.3274	3.3984	3.4429	3.5236	3.6684	3.7120
LSSVR-PF in [26]		2.4915	2.6860	3.1510	3.1835	3.4320	3.3845	3.3690	3.5250	3.7554	3.8613
ToA-PF in [28]		5.3947	5.1016	4.8245	4.4975	4.5450	4.4157	4.3370	4.1996	4.2199	4.2943

**Table 3:** The average localization errors in the excessive noise condition.

	Time Step (s)	k=10	k=20	k=30	k=40	k=50	k=60	k=70	k=80	k=90	k=100
Method											
SVL-PF in this paper		3.4576	3.5440	3.5353	3.5832	3.4888	3.4695	3.4276	3.4292	3.4305	3.4523
Consensus Estimation in [14]		4.1671	4.6963	5.1126	5.9764	5.8122	5.7013	5.6783	5.6683	5.7145	5.9295
LSSVR-PF in [26]		5.2951	5.5807	5.5747	6.1289	6.1254	6.6172	7.1446	7.5155	7.8664	8.2197
ToA-PF in [28]		4.9028	4.6084	5.3006	5.8910	6.1165	6.3656	6.4334	6.4526	6.3611	6.5046

## Abbreviations

The following abbreviations are used in this manuscript:

UASNs	underwater acoustic sensor networks
SVM	support vector machine
LSSVR	least square support vector regression
ToA	time of arrival
AUV	Autonomous Underwater Vehicle
SVL-PF	support vector learning-based particle filter
LSSVR-PF	least-square support vector regression-based particle filter
ToA-PF	ToA-based particle filter
